# Efficacy and safety of different JAK inhibitors in the treatment of alopecia areata: a network meta-analysis

**DOI:** 10.3389/fimmu.2023.1152513

**Published:** 2023-04-17

**Authors:** Dongfan Wei, Yi Chen, Yuqing Shen, Bo Xie, Xiuzu Song

**Affiliations:** ^1^ Department of Dermatology, Affiliated Hangzhou Dermatology Hospital, Zhejiang University School of Medicine, Hangzhou, China; ^2^ Department of Dermatology, Hangzhou Third People’s Hospital, Zhejiang Chinese Medical University, Hangzhou, China; ^3^ Department of Dermatology, Hangzhou Third People’s Hospital, Affiliated Hangzhou Dermatology Hospital, Zhejiang University School of Medicine, Hangzhou, China

**Keywords:** alopecia areata, JAK inhibitors, baricitinib, ruxolitinib, tofacitinib, network meta-analysis

## Abstract

**Background:**

Alopecia areata (AA) is an immune disease characterized by non-scarring hair loss. With the widespread application of JAK inhibitors in immune-related diseases, attention is being given to their role in the treatment of AA. However, it is unclear which JAK inhibitors have a satisfactory or positive effect on AA. This network meta-analysis aimed to compare the efficacy and safety of different JAK inhibitors in the treatment of AA.

**Methods:**

The network meta-analysis was performed according to the PRISMA guidelines. We included randomized controlled trials as well as a small number of cohort studies. The differences in efficacy and safety between the treatment and control groups were compared.

**Results:**

Five randomized controlled trials, two retrospective studies, and two prospective studies involving 1689 patients were included in this network meta-analysis. In terms of efficacy, oral baricitinib and ruxolitinib significantly improved the response rate of patients compared to placebo [MD = 8.44, 95% CI (3.63, 19.63)] and [MD = 6.94, 95% CI, (1.72, 28.05)],respectively. Oral baricitinib treatment significantly improved the response rate compared to non-oral JAK inhibitor treatment [MD=7.56, 95% CI (1.32,43.36)]. Oral baricitinib, tofacitinib, and ruxolitinib treatments significantly improved the complete response rate compared to placebo [MD = 12.21, 95% CI (3.41, 43.79)], [MD = 10.16, 95% CI (1.02, 101.54)], and [MD = 9.79, 95% CI, (1.29, 74.27)], respectively. In terms of safety, oral baricitinib, tofacitinib, and ruxolitinib treatments significantly reduced treatment-emergent adverse event rates compared with conventional steroid treatment [MD = 0.08, 95% CI (0.02, 0.42)], [MD = 0.14, 95% CI (0.04, 0.55)], and [MD = 0.35, 95% CI, (0.14, 0.88)], respectively.

**Conclusion:**

Oral baricitinib and ruxolitinib are excellent options for the treatment of AA owing to their good efficacy and safety profiles. In contrast, non-oral JAK inhibitors do not appear to have satisfactory efficacy in treating AA. However, further studies are required to verify the optimal dose of JAK inhibitors for AA therapy.

## 1 Introduction

Alopecia areata (AA) is a common, non-scarring, autoimmune hair loss disorder mediated by the attack on hair follicles (1). The prevalence of AA in China is approximately 0. 27%. However, its prevalence can vary from 0.1% to 6.9% depending on the population and study area (2). Common clinical manifestations include sudden round alopecia spots, mostly without conscious symptoms, and a few patients may have mild scalp itching or tightness. Those with a small area of alopecia tend to self-manage the condition. However, some individuals experience more severe symptoms, such as alopecia totalis (AT) (full loss of hair on the scalp) and alopecia universalis (AU) (full loss of hair on the scalp and body) (3). Persistent AA and its variants can have devastating effects on the mental health and quality of life of the patient (4).

Clinically, topical, intralesional, and systemic corticosteroids, glycyrrhizin, minoxidil, diphenylcyclopropenone, and systemic agents such as methotrexate are used to alleviate AA in patients (3). Unfortunately, there is currently no cure for AA. Further, corticosteroid therapy, the most commonly used strategy in clinical practice, is associated with side effects such as acne, weight gain, and endocrine disorders. Owing to this, more effective and safe treatment options are urgently needed for the treatment of AA.

The Janus kinase/signal transducer and activator of transcription (JAK/STAT) signaling pathway is a key communication hub for cell function (5). Janus kinase inhibitors are a class of small-molecule compounds that can block one or more intracellular tyrosine kinases in the JAK-STAT signaling pathway, including JAK1, JAK2, JAK3, and tyrosine kinase 2 (TYK2). They thus block various cytokine and inflammatory pathways and induce immune suppression (6). A recent study (7) showed that JAK inhibitors can block the T lymphocyte-mediated immune response in hair follicles, promote the formation of hair follicle stem cells, and trigger angiogenesis, both of which occur during the hair growth phase. JAK inhibitors can also accelerate the transition of hair follicles from the telogen phase to the anagen phase (8).

Many clinical studies have shown that the use of JAK inhibitors to treat AA has achieved satisfactory outcomes with an acceptable or tolerable side effect profile. To date, no meta-analyses comparing the efficacy of different JAK inhibitors in the treatment of AA have been published. Therefore, this study aimed to indirectly compare the efficacy and safety of various JAK inhibitors in the treatment of AA using a network meta-analysis to lay a solid foundation for the clinical treatment of the condition.

## 2 Materials and methods

### 2.1 Retrieval strategy

The network meta-analysis was performed according to the Preferred Reporting Items for Systematic Reviews and Meta-Analyses (PRISMA) extension of the Network Meta-check Analysis List. Electronic searches were performed across four electronic databases, viz. PubMed, Embase, Web of Science, and the Cochrane Library, from inception until January 2023.

The search strategy for the aforementioned databases is summarized in **Supplementary Material**.

### 2.2 Inclusion and exclusion criteria

#### 2.1.1 Inclusion standards

(1) Study type. Randomized controlled trials (RCT), prospective trials, and retrospective studies on JAK inhibitors for the treatment of patients with AA. The studies included both control and experimental groups.
(2) Participants Definitively diagnosed with AA/AT/AU.
(3) Intervention measures. The control group was treated with a placebo or conventional steroid therapy. The experimental groups were treated with oral or topical JAK inhibitors. At least two trials were required to confirm the efficacy of the same AA treatment regimen. The included trials provided efficacy (scalp hair regrowth rate) and safety (adverse events) outcomes.

#### 2.1.2 Exclusion standards

(1) Studies including patients with only eyelash and eyebrow involvement, but no scalp involvement.
(2) Inability to extract data or missing data.
(3) Case reports or case series (fewer than six cases); abstracts; conference presentations; editorials; reviews; or expert opinions.

### 2.3 Data extraction

Two researchers independently screened for inclusion and crosschecked the results. Differences between the researchers were resolved through consultation.

Data extraction standards included the following: main author, study type, sample size, sex ratio, mean age, intervention measures in the experimental group, intervention measures in the control group, and initial SALT score.

The main treatment outcomes were good response (defined as a > 50% decrease in SALT score), complete response (defined as a > 90% decrease in SALT score), and percent change from baseline in the SALT score.

Safety outcomes included the rate of adverse events.

### 2.4 Bias risk assessment

The quality of the included studies was evaluated according to the Cochrane 5.1 risk of bias assessment tool, which includes seven aspects: the random sequence method; whether the assignment was hidden; whether the implementation process was blinded; whether the evaluators were blinded; whether there were missing data; whether the outcome indicators were selectively reported; and whether there were other biases. We divided the above quality assessment into three grades: “unclear” (lack of relevant data), “low” (low risk), and “high” (high risk). The risk profile of each item was determined, and the results were visualized using th Review Manager 5.4.1 software. For non-randomized control trials (RCTs), the Newcastle–Ottawa Scale (NOS) was used (9, 10). The evaluation is comprised of three domains: selection, comparability, and outcome. The NOS adopts a semi-quantitative principle of the star system to evaluate the quality of literature, with a full score being nine stars.

### 2.5 Statistical analysis

RevMan v5.4 was used to evaluate the quality of the literature, and the risk of bias map was determined. Stata17.0 was used for the traditional meta-analysis. The odds ratio (OR) and 95% confidence interval (CI) were the effect size indicators for dichotomous variables (good response rate, complete response rate, and incidence of adverse reactions). The mean difference (MD) and 95% CI were regarded as effect size indicators for continuous variables (percentage change in the SALT score from baseline).

I2 and P values were used to evaluate the heterogeneity between the results of the studies. P≥ 0.05 or I2≤ 50% indicated that the heterogeneity between the studies was small, and the fixed effect model was used to combine the effect size. When P < 0.05 or I2 > 50%, substantial heterogeneity was considered to exist (1). Subgroup analysis was used to determine the source of heterogeneity, and sensitivity analysis was used to judge the stability and strength of the results. If the source of heterogeneity could not be found, the random-effects model was used to pool the effect size, or the meta-analysis was abandoned and only descriptive analysis was performed. Stata17.0 software, based on the frequency method, was used to conduct a network meta-analysis in which group commands were used for network analysis of the study outcome measures. Data processing, network evidence plots, funnel plots, surface under the cumulative ranking curve (SUCRA) ranking, and forest plots were generated. SUCRA was used to visually analyze the advantages and disadvantages of the interventions for each outcome indicator. SUCRA values range from 0 to 100, and the closer it is to 100, the better the intervention (11). A comparison-corrected funnel plot was drawn to evaluate whether there was a small-sample effect or publication bias.

## 3 Results

### 3.1 Literature search and screening

**Figure 1:**
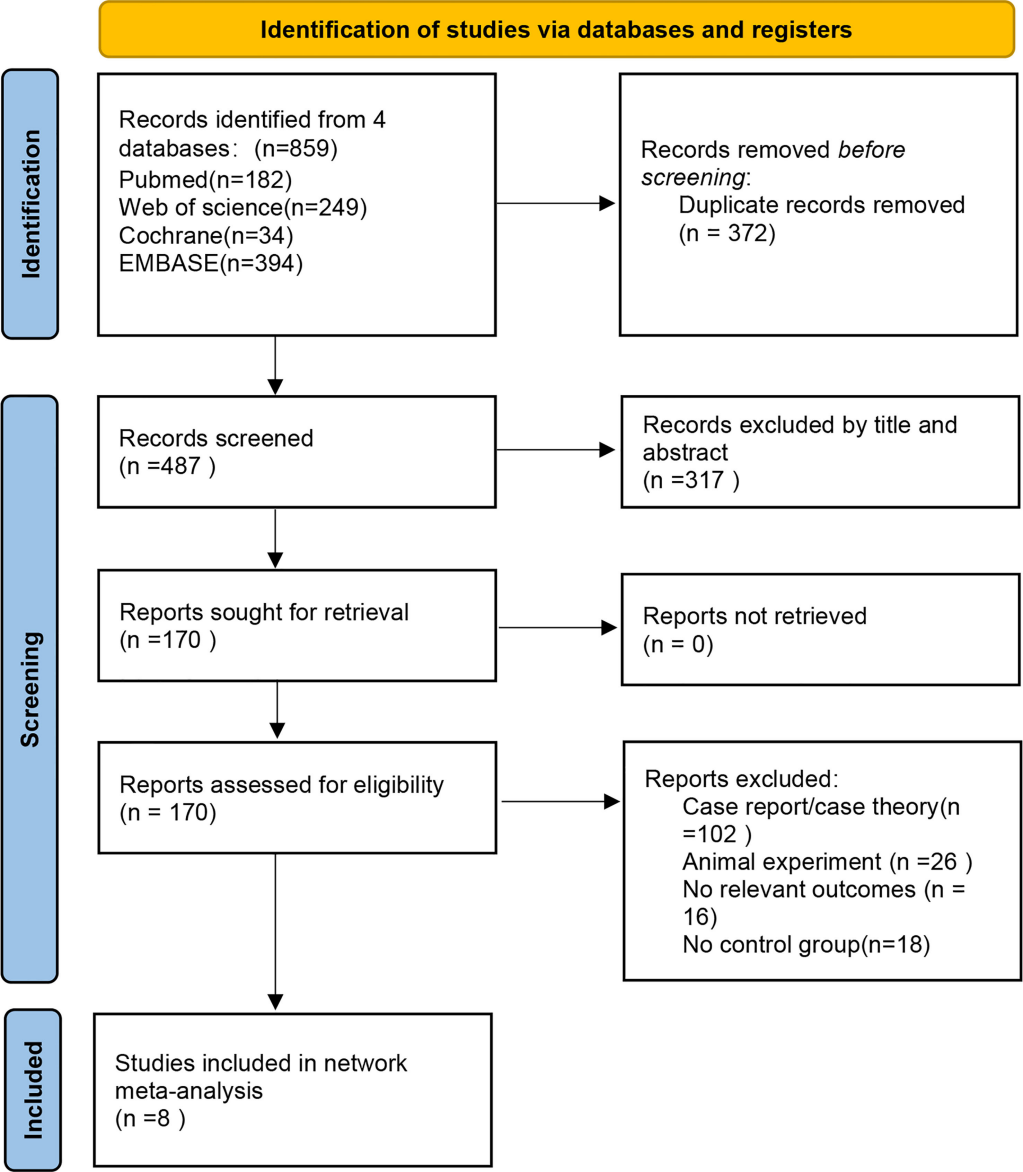
Flow diagram.

A total of 859 relevant studies were identified after preliminary retrieval. After excluding duplicates and unrelated articles, 487 potentially relevant articles were identified. After reading titles and abstracts, 25 articles were included in the analysis. After further reading the full texts, articles without control group studies or relevant outcome indicators were excluded, and eight articles (12–19) made the final selection, comprising five RCTs (one (18) involved two RCTs), two retrospective studies, and two prospective studies (**Figure 1**).

### 3.2 Basic characteristics

The eight studies included 1689 patients. **Table 1** provides basic information on the relevant literature.

**Table 1:** Demographic and clinical characteristics of the patients at baseline.

author (year)	study type	N	gender (F-M)	age (mean,SD,year)	treatment	initial SALT score (%)	treatment duration
Wenxin Zhang (2022)	retrospective study	C:18	10-8	30.3 (6.83)	steroids treatment PO	58.0 (16.71)	7.1 (4.2–14.4)months
		T:23	15-8	35.9 (3.78)	5mg Tofacitinib PO QD/BID	60.0 (10.37)	9.3 (5.3–12.9)months
Jung-Won Shin (2019)	retrospective study	C:26	12-14	25.7 (15.15)	steroids treatment PO	96.3 (5.05)	6 months
		T:18	11-7	20.9 (12.63)	5mg Tofacitinib PO BID/TID	97.3 (4.67)	6 months
V. W. Y. Lai (2021)	prospective study	C:2	1-1	34 (5)	steroids treatment PO	41.8 (19.75)	12 weeks
		T:18	14-4	45.1 (15.28)	5mg Tofacitinib sublingual BID	86.0 (23.30)	12 weeks
N. Almutairi (2019)	prospective study	C:38	17-21	35.5 (13.8)	Ruxolitinib 20 mg PO BID	94.2 (12.76)	24 weeks
		T:37	15-22	47.4 (16.1)	Tofacitinib 5 mg PO BID	93.4 (14.03)	24 weeks
E. A. Olsen (2020) Bpart	RCT	C:39	27-12	42.4 (12.5)	placebo	59.0 (25.3)	24 weeks
		T:39	24-15	44.3 (12.5)	1.5% ruxolitinib cream	59.9 (29.4)	24 weeks
B. King (2021)	RCT	C:28	16-12	40.5 (14.2)	placebo	90.0 (15.7)	36 weeks
		T1:27	23-4	42.5 (13.8)	2mg baricitinib PO QD	86.1 (19.3)	36 weeks
		T2:27	25-2	42.4 (14.9)	4mg baricitinib PO QD	83.4 (17.5)	36 weeks
B. King (2022) BRAVE-AA1	RCT	C:189	109-80	37.4 (12.9)	placebo	84.7 (17.8)	36 weeks
		T1:184	109-75	38.0 (12.8)	2mg baricitinib PO QD	86.8 (18.0)	36 weeks
		T2:281	165-116	36.3 (13.3)	4mg baricitinib PO QD	85.3 (18.2)	36 weeks
B. King (2022) BRAVE-AA2	RCT	C:156	98-58	37.1 (12.4)	placebo	85.0 (17.8)	36 weeks
		T1:156	103-53	39.0 (13.0)	2mg baricitinib PO QD	85.6 (18.1)	36 weeks
		T2:234	144-90	38.0 (12.7)	4mg baricitinib PO QD	84.8 (18.1)	36 weeks
B. King (2022)	RCT	C:44	29-15	37.8 (13.50)	placebo	86.8 (18.39)	24 weeks
		T1:30	22-8	35.7 (11.01)	4mg CTP-543* PO QD	88.8 (16.19)	24 weeks
		T2:38	26-12	37.3 (14.18)	8mg CTP-543 PO QD	89.1 (16.41)	24 weeks
		T3:37	28-9	35.8 (12.37)	12mg CTP-543 PO QD	87.3 (18.74)	24 weeks

CTP-543: Ruxolitinib deuteride.

Among the five RCTs included, three compared oral baricitinib with a placebo, one compared oral CTP-543 (deuterated ruxolitinib) with a placebo, and one compared topical ruxolitinib ointment with a placebo. Two retrospective studies comparing oral tofacitinib with conventional steroid therapy were included. Between the two prospective studies included, one compared sublingual tofacitinib with cyclosporine and the other compared oral tofacitinib with oral ruxolitinib.

### 3.3 Bias risk assessment

**Figure 2:**
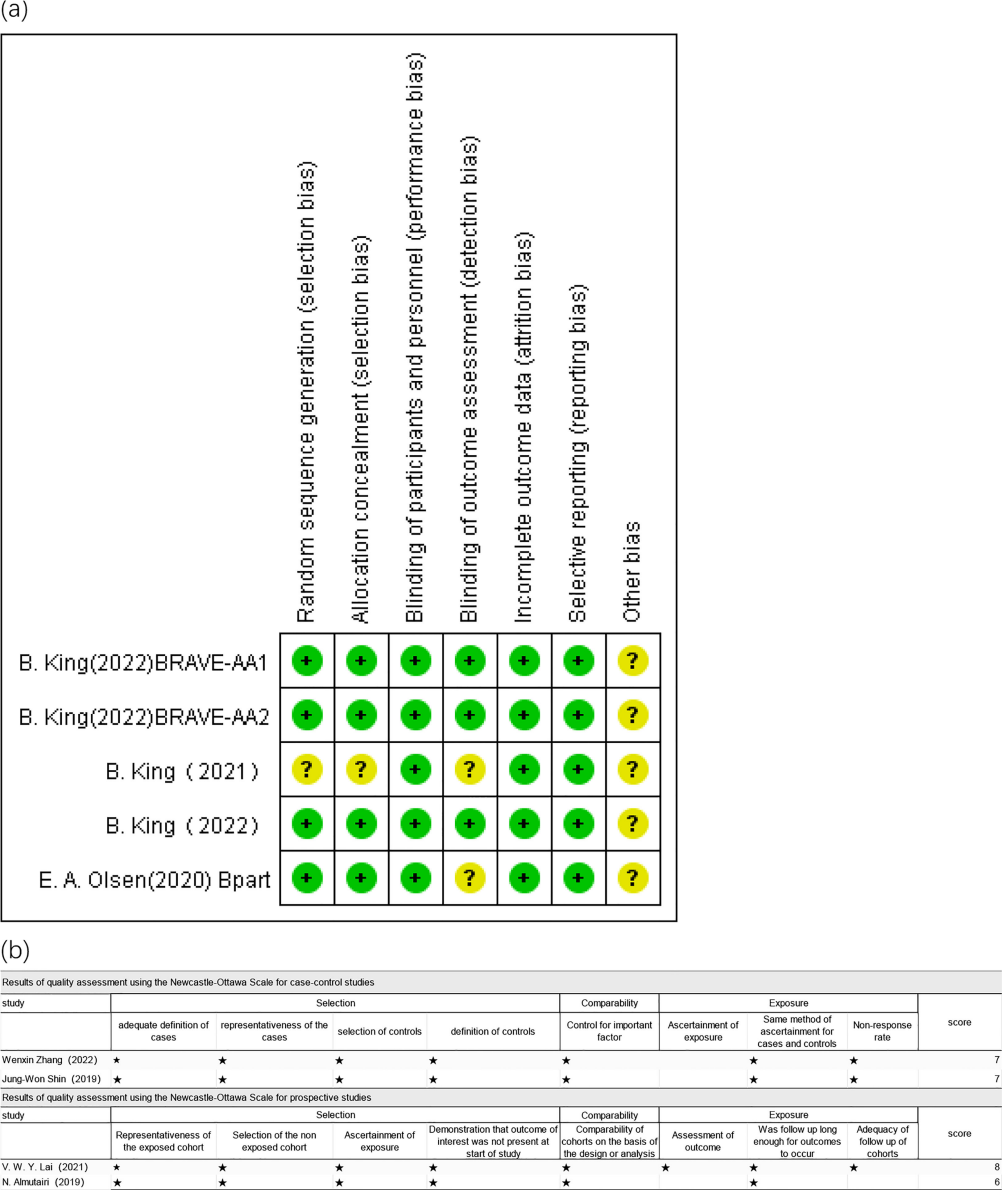
Risk of bias in **(A)** included RCTs and **(B)** included non-RTCs.

**Figure 2** depicts the risk of bias assessment of the included studies.

### 3.4 Efficacy outcome

#### 3.4.1 Good response rate

**Figure 3:**
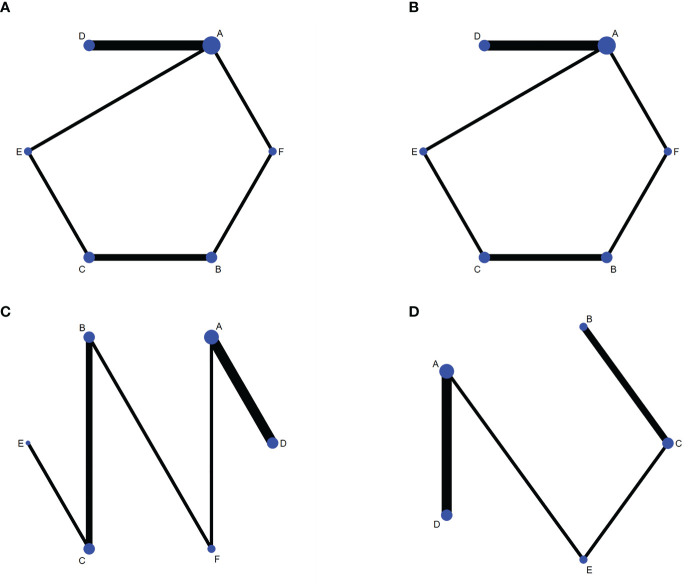
Network diagrams of outcome indicators. **(A)** good response rate; **(B)** complete response rate; **(C)** percent change form baseline in SALT scorer; **(D)** TEAE (treatment emergent adverse events) rate. A: placebo treatment B: conventional steroid therapy C: any dose of oral tofacitinib treatment D: any dose of oral baricitinib treatment E: any dose of oral ruxolitinib treatment F: nonoral JAK inhibitor treatment.

**Figure 4:**
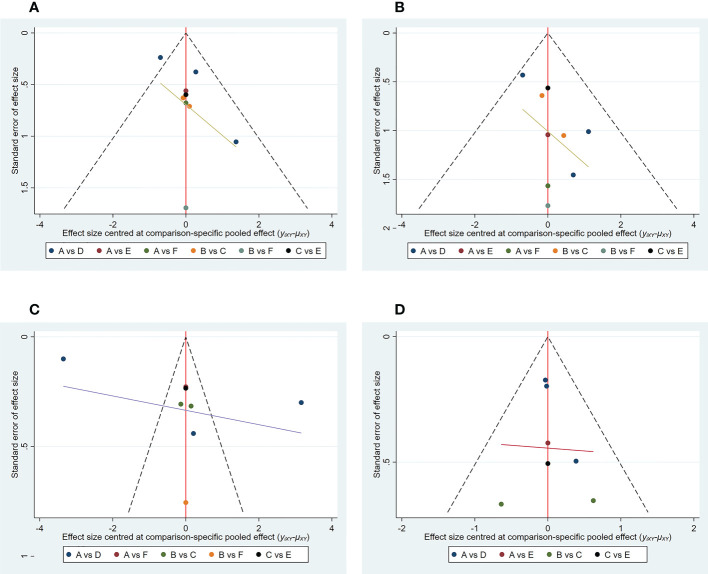
Funnel plot of outcome indicators. **(A)** good response rate; **(B)** complete response rate; **(C)** percent change from baseline in SALT score; **(D)** TEAE (treatment emergent adverse events) rate. A: placebo treatment B: conventional steroid therapy C: any dose of oral tofacitinib treatment D: any dose of oral baricitinib treatment E: any dose of oral ruxolitinib treatment F: nonoral JAK inihibitor treatment.

**Figure 5:**
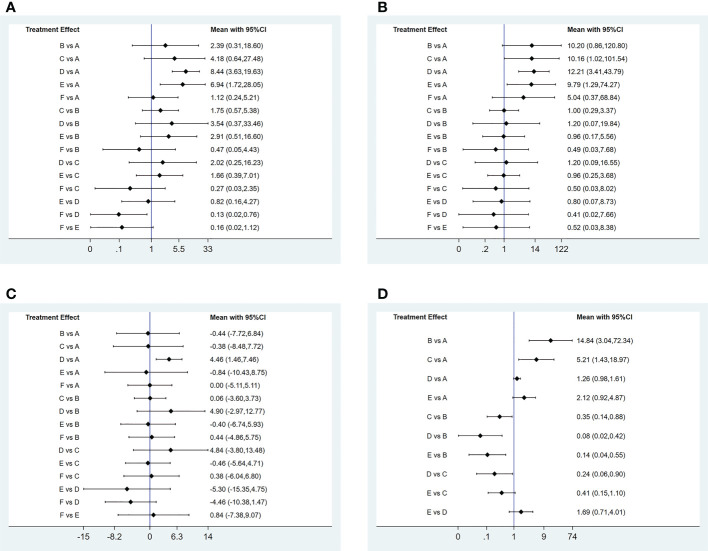
Pairwise comparison forest graph of outcome indicators. **(A)** good response rate; **(B)** complete response rate; **(C)** percent change from baseline in SALT score; **(D)** TEAE (treatment emergent adverse events) rate. A: placebo treatment B: conventional steroid therapy C: any dose of oral tofocitinib treatment D: any dose of oral baricitinib treatment E: any dose of oral ruxolitinib treatment : nonoral JAK inihibitor treatment.

**Figure 6:**
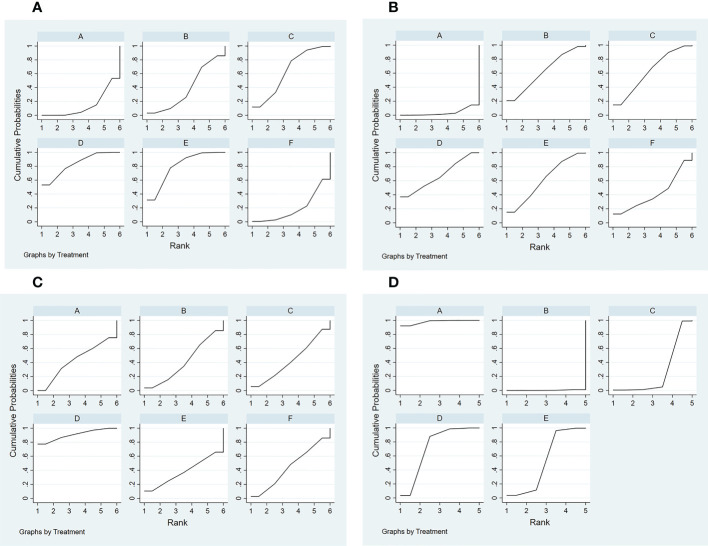
Curve diagram of SUCRA of outcome indicators. **(A)** good response rate; **(B)** complete response rate; **(C)** percent change from baseline in SALT score; **(D)** TEAE (treatment emergent adverse events) rate. A: placebo treatment B: conventional steroid therapy C: any dose of oral tofacitinib treatment D: any dose of oral baricitinib treatment E: any dose of oral ruxolitinib treatment F: nonoral JAK inhibitor treatment.

1) Evidence network. The sizes of the dots and lines in the network are proportional to the number of studies, with thicker lines indicating more studies comparing JAK inhibitors to other therapies. The results show that the number of studies comparing oral baricitinib with placebo was the largest, followed by those comparing oral tofacitinib with conventional hormonal therapy. Relatively few studies have, to date, compared the non-oral JAK inhibitor ruxolitinib with other treatments (**Figure 3A**).
2) Publication bias. The funnel plot results show that most scattering points are located on both sides of the vertical line. Some studies fall outside the funnel plot, indicating that there may have been publication bias or small sample effects in the included literature (**Figure 4A**).
3) Network meta-analyses. Network comparisons were performed for six treatment modalities, three of which were statistically significant. Compared with placebo, the MD and 95% CI of any doses of oral baricitinib and ruxolitinib were 8.44 and [3.63, 19.63] and 6.94 and [1.72, 28.05], respectively. Compared to non-oral JAK inhibitor treatment, the MD and 95% CI of any dose of oral baricitinib treatment was 7.56 and [1.32, 43.36], respectively (**Figure 5A** and **Table 2A**).
4) SUCRA probability ranking. The probability of a high or low response rate to JAK inhibitor treatment ranked according to the SUCRA (**Figure 6A**) was as follows: any dose of oral baricitinib treatment (83.6%) > any dose of oral ruxolitinib treatment (80.1%) > any dose of oral tofacitinib treatment (63.4%) > conventional steroid therapy (38.8%) > nonoral JAK inhibitor treatment (19.4%) > placebo (14.5%).

**Table 2A:** Matrix of good response rate after different JAKi treatment(shown as SMD and 95% Cls).

	D	E	C	B	F	A
D	1	0.82 (0.16,4.27)	0.50 (0.06,3.99)	0.28 (0.03,2.67)	0.13 (0.02,0.76)	0.12 (0.05,0.28)
E	1.22 (0.23,6.31)	1	0.60 (0.14,2.55)	0.34 (0.06,1.96)	0.16 (0.02,1.12)	0.14 (0.04,0.58)
C	2.02 (0.25,16.23)	1.66 (0.39,7.01)	1	0.57 (0.19,1.75)	0.27 (0.03,2.35)	0.24 (0.04,1.57)
B	3.54 (0.37,33.46)	2.91 (0.51,16.60)	1.75 (0.57,5.38)	1	0.47 (0.05,4.43)	0.42 (0.05,3.27)
F	7.56 (1.32,43.36)	6.22 (0.90,43.17)	3.75 (0.43,33.01)	2.14 (0.23,20.24)	1	0.90 (0.19,4.18)
A	8.44 (3.63,19.63)	6.94 (1.72,28.05)	4.18 (0.64,27.48)	2.39 (0.31,18.60)	1.12 (0.24,5.21)	1

MD, standard mean difference.

Red texts indicated that there were statistical difference between groups corresponding to horizontal and vertical coordinates.

#### 3.4.2 Complete response rate

1) Evidence network. The sizes of the dots and lines in the network are proportional to the number of studies, with thicker lines indicating more studies comparing JAK inhibitors to other therapies. The results show that the number of studies comparing oral baricitinib with placebo was the largest, followed by those comparing oral tofacitinib with conventional hormonal therapy. Relatively few studies have, to date, compared the non-oral JAK inhibitor ruxolitinib with other treatments (**Figure 3B**).
2) Publication bias. The funnel plot results show that most scattering points are located on both sides of the vertical line. Some studies fall outside the funnel plot, indicating that there may have been publication bias or small sample effects in the included literature (**Figure 4B**).
3) Network meta-analyses. Network comparisons were performed for six treatment modalities, three of which were statistically significant. Compared with the placebo, the MD and 95% CI of any dose of oral baricitinib, tofacitinib, and ruxolitinib treatments were 12.21 and [3.41,43.79], 10.16 and [1.02,101.54], and 9.79 and [1.29,74.27], respectively (**Figure 5B** and **Table 2B**).
4) SUCRA probability ranking. The probability of a high or low complete response rate for JAK inhibitor treatment was ranked according to the SUCRA (**Figure 6B**) as follows: any dose of oral baricitinib (67.6%) > conventional steroid therapy (62.9%) > any dose of oral tofacitinib (62.7%) > any dose of oral ruxolitinib (61.2%) > non-oral JAK inhibitor (41.9%) > placebo (3.7%).

**Table 2B:** Matrix of complete response rate after different JAKi treatment(shown as SMD and 95% Cls).

	D	B	C	E	F	A
D	1	0.84 (0.05,13.85)	0.83 (0.06,11.44)	0.80 (0.07,8.73)	0.41 (0.02,7.66)	0.08 (0.02,0.29)
B	1.20 (0.07,19.84)	1	1.00 (0.29,3.37)	0.96 (0.17,5.56)	0.49 (0.03,7.68)	0.10 (0.01,1.16)
C	1.20 (0.09,16.55)	1.00 (0.30,3.41)	1	0.96 (0.25,3.68)	0.50 (0.03,8.02)	0.10 (0.01,0.98)
E	1.25 (0.11,13.58)	1.04 (0.18,6.04)	1.04 (0.27,3.96)	1	0.52 (0.03,8.38)	0.10 (0.01,0.77)
F	2.42 (0.13,44.89)	2.02 (0.13,31.44)	2.01 (0.12,32.51)	1.94 (0.12,31.58)	1	0.20 (0.01,2.70)
A	ss12.21 (3.41,43.79)	10.20 (0.86,120.80)	10.16 (1.02,101.54)	9.79 (1.29,74.27)	5.04 (0.37,68.84)	1

MD, standard mean difference.

Red texts indicated that there were statistical difference between groups corresponding to horizontal and vertical coordinates.

#### 3.4.3 Percent change from baseline in SALT score

1) Evidence network. The sizes of the dots and lines in the network are proportional to the number of studies, with thicker lines indicating more studies comparing JAK inhibitors to other therapies. The results show that the number of studies comparing oral baricitinib with placebo was the largest, followed by those comparing oral tofacitinib with conventional hormonal therapy. Relatively few studies, to date, have compared the non-oral JAK inhibitor ruxolitinib with other treatments (**Figure 3C**).
2) Publication bias. The funnel plot results show that most scattering points are located on both sides of the vertical line. Some studies fall outside the funnel plot, indicating that there may have been publication bias or small sample effects in the included literature (**Figure 4C**).
3) Network meta-analyses. Network comparisons were performed for six treatment modalities, one of which was statistically significant. Compared with the placebo, the MD and 95% CI of any dose of oral baricitinib treatment was 86.50 and [4.31, 1737.70], respectively (**Figure 5C** and **Table 2C**).
4) SUCRA probability ranking. In accordance with the SUCRA (**Figure 6C**), the percent change from baseline in SALT score of JAK inhibitor treatment was ranked from high to low as follows: any dose of oral baricitinib (90.5%) > non-oral JAK inhibitor (44.8%) > placebo (43.0%) > any dose of oral tofacitinib (43.0%) > conventional steroid therapy (40.8%) > any dose of oral ruxolitinib (37.9%).

**Table 2C:** Matrix of percent change in SALT scPercent change in SALT score after different JAKi treatment(shown as SMD and 95% Cls).

	D	F	A	C	B	E
D	D	0.01 (0.00,4.35)	0.01 (0.00,0.23)	0.01 (0.00,44.76)	0.01 (0.00,19.53)	0.00 (0.00,115.44)
F	86.18 (0.23,32299.37)	F	1.00 (0.01,165.21)	0.68 (0.00,418.41)	0.64 (0.00,129.43)	0.43 (0.00,1611.20)
A	86.50 (4.31,1737.70)	1.00 (0.01,166.45)	A	0.68 (0.00,2261.72)	0.64 (0.00,932.64)	0.43 (0.00,6315.09)
C	126.44 (0.02,715504.00)	1.47 (0.00,900.71)	1.46 (0.00,4832.74)	C	0.94 (0.02,36.77)	0.63 (0.00,111.57)
B	134.31 (0.05,352299.13)	1.56 (0.01,314.40)	1.55 (0.00,2248.65)	1.06 (0.03,41.49)	B	0.67 (0.00,377.70)
E	200.61 (0.01,4.65e+06)	2.33 (0.00,8731.76)	2.32 (0.00,33970.69)	1.59 (0.01,280.89)	1.49 (0.00,842.68)	E

MD, standard mean difference.

Red texts indicated that there were statistical difference between groups corresponding to horizontal and vertical coordinates.

### 3.5 Safety outcome

#### 3.5.1 Treatment emergent adverse event rate

1) Evidence network. The sizes of the dots and lines in the network are proportional to the number of studies, with thicker lines indicating more studies comparing JAK inhibitors to other therapies. The results show that the number of studies comparing oral baricitinib with placebo was the largest, followed by those comparing oral tofacitinib with conventional hormonal therapy. Relatively few studies have, to date, compared ruxolitinib with other treatments (**Figure 3D**).
2) Publication bias. The funnel plot results show that most scattering points are located on both sides of the vertical line (**Figure 4D**).
3) Network meta-analyses. Network comparisons were performed for five treatment modalities, six of which were statistically significant. Compared with conventional steroid therapy, the MD and 95% CI of placebo treatment for any dose of oral baricitinib treatment, any dose of oral tofacitinib treatment, and any dose of oral ruxolitinib treatment were 0.07 and [0.01,0.33], 0.08 and [0.02,0.42], 0.14 and [0.04,0.55], and 0.35 and [0.14,0.88)], respectively. Compared with any dose of oral tofacitinib, the MD and 95% CI of placebo treatment and any dose of oral baricitinib were 0.19 and [0.05, 0.70] and 0.24 and [0.06, 0.90], respectively (**Figure 5D** and **Table 2D**).
4) SUCRA probability ranking. Based on the SUCRA (**Figure 6D**), the TEAE rate of JAK inhibitor treatment was ranked by likelihood from low to high as follows: placebo (97.9%) > any dose of oral baricitinib (72.5%) > any dose of oral ruxolitinib (52.8%) > any dose of oral tofacitinib (26.4%) > conventional steroid therapy (0.3%).

**Table 2D:** Matrix of good response rate after different JAKi treatment(shown as SMD and 95% Cls).

	A	D	E	C	B
A	A	1.26 (0.98,1.61)	2.12 (0.92,4.87)	5.21 (1.43,18.97)	14.84 (3.04,72.34)
D	0.80 (0.62,1.02)	D	1.69 (0.71,4.01)	4.14 (1.11,15.45)	11.81 (2.38,58.68)
E	0.47 (0.21,1.08)	0.59 (0.25,1.41)	E	2.45 (0.91,6.61)	7.00 (1.82,26.96)
C	0.19 (0.05,0.70)	0.24 (0.06,0.90)	0.41 (0.15,1.10)	C	2.85 (1.14,7.12)
B	0.07 (0.01,0.33)	0.08 (0.02,0.42)	0.14 (0.04,0.55)	0.35 (0.14,0.88)	B

MD, standard mean difference.

Red texts indicated that there were statistical difference between groups corresponding to horizontal and vertical coordinates.

#### 3.5.2 Adverse event analysis

Details of the adverse events associated with the different treatments are shown in **Table 3**. Acneiform eruption, hyperlipidemia, upper respiratory infection, and headache appear to be the most common adverse reactions to oral JAK inhibitors for the treatment of AA (**Table 3**). In the included trials, one cancer event was reported in the oral baricitinib group. However, similar events were reported in the placebo group, with no statistically significant difference. Therefore, we believe that there is no clear link between the occurrence of neoplastic events and the treatment of AA with oral JAK inhibitors. A small proportion of patients discontinued JAK inhibitors because of severe side effects; most of these patients were in the high-dose oral JAK inhibitor group.

**Table 3:** Summary of adverse events.

Complication	Retrospective study		Prospective study		RCT			
	Wenxin Zhang(2022)	Jung-Won Shin(2019)	V. W. Y. Lai(2021)	N. Almutairi(2019)	B. King (2021)	B. King (2022)BRAVE-AA1	B. King (2022)BRAVE-AA2	B. King (2022)
Upper respiratory infection	2/23	–	–	4/75	9/54	30/463	27/388	11/103
Nasopharyngitis	–	1/18	–	1/75	–	33/463	17/388	15/103
Urinary tract infection	1/23	–	–	9/75	–	9/463	23/388	–
Tuberculosis	0/23	–	–	0/75	0/54	0/463	0/388	–
Liver enzyme abnormalities	2/23	–	–	5/75	–	–	–	–
Hypocytosis	–	1/18	–	4/75	–	–	–	–
Blood creatine kinase increased	–	–	–	–	–	18/463	7/388	6/103
Hyperlipidemia	2/23	–	–	3/75	–	84/463	81/388	4/103
Headache	–	–	–	4/75	–	22/463	33/388	22/103
Nausea	–	–	–	–	4/54	–	–	9/103
Urticaria	1/23	2/18	–	–	–	–	–	–
Diarrhea	–	1/18	–	2/75	–	–	–	4/103
Non-specific Infection	–	1/18	–	3/75	2/54	0/463	0/388	–
Herpes	–	–	–	3/75	6/54	8/463	14/388	–
Acneiform eruption	6/23	–	–	5/75	5/54	26/463	20/388	14/103

## 4 Discussion

AA is a T cell-mediated autoimmune disease phenotypically characterized by alopecia and histologically by T cell infiltration around hair follicles (20). Steroid therapy is the most widely used treatment strategy for this condition; however, with the deepening of the mechanistic understanding of the potential key T-cell inflammatory pathways in AA, IFN-γ has been identified as an important pathogenic cytokine (21), and its related JAK-STAT pathway has become a new therapeutic target. Owing to this, JAK inhibitors have become a new option for the clinical treatment of AA. However, no clear conclusion has been reached as to which JAK inhibitor has the best therapeutic effect against AA. This study is the first to use a network meta-analysis to compare the efficacy and safety of different JAK inhibitors in the treatment of AA with the aim of providing a reference for the clinical use of JAK inhibitors and the design of subsequent related studies.

The efficacy outcomes in this study demonstrated that oral baricitinib and ruxolitinib significantly increased the frequency of good responses compared to placebo. Baricitinib has been proposed as a dual JAK1/JAK2 inhibitor given its strong binding interactions with both proteins. Ruxolitinib is generally considered a JAK1/JAK2 inhibitor that inhibits both γc family cytokine signaling (JAK1/JAK3) and IFNγ signaling (JAK1/JAK2). We hypothesized that baricitinib and ruxolitinib would display good efficacy, which may be related to their inhibition of IFNγ production. JAK inhibition regulates the activation of key hair follicle populations such as hair embryos and increases the induction rate of cultured human hair papilla cells by controlling molecular signals enriched in intact, fully induced hair papillae (22).

In our analysis, we found no significant difference in the frequency of good response between conventional steroid therapy and placebo, probably because most patients enrolled in the trial chose other treatments after the failure of conventional treatment. Therefore, we hypothesize that oral JAK inhibitors may be one of the most effective treatments currently available for AA, especially in patients for whom conventional steroid therapy has failed.

We found that topical JAK inhibitors had no significant therapeutic effects. Bokhari et al. (23), in a prospective, placebo-controlled, double-blind phase I study, proposed that topical JAK inhibitors may become a new strategy for the treatment of AA. We postulate that this contradiction with our current findings may be because the sample size of the previous study was too small, allowing the spontaneous remission of AA and placebo effects to be incorrectly attributed to the use of topical JAK inhibitors, as reflected in a study by Olsen et al. (16).

Despite the advantages of oral JAK inhibitors over conventional steroid therapy in terms of good response rates, we found no significant difference in the complete response rate between the commonly used JAK inhibitor, tofacitinib, and conventional steroid therapy. On the one hand, the pathogenesis of AA is not completely clear, and steroid-insensitive immune cell lines such as innate lymphocytes (ILCs) may be involved in the occurrence and development of inflammation in AA (24, 25), making conventional steroid therapy an unsuitable strategy to completely cure AA. Nonetheless, AA is often combined with several other chronic inflammatory skin diseases such as atopic dermatitis and vitiligo, which are both chronic inflammatory diseases dominated by inflammatory factors. When AA patients suffer from other immune-related diseases, there is often excessive activation of Th2, which causes the increase of IgE. Therefore, a combination of targeted drugs (such as dupilumab) is required (26, 27). In addition, the neuroendocrine mechanism of mental stress (28), genetic background (29), and other factors have an impact on the progression of AA. The complex pathogenesis of AA remains to be explored to elucidate direct and more effective therapeutic targets.

In addition, a variety of JAK inhibitors are being tested in clinical trials for the treatment of alopecia areata. King et al. has performed a phase 2a randomized, placebo-controlled study to evaluate the efficacy and safety of the oral JAK inhibitors ritlecitinib(JAK3 selective inhibitor) and brepocitinib(JAK1/TYK2 inhibitor) in AA (30). In this clinical trial, we observed significant improvements in both good response rate and complete response rate in patients treated with ritlecitinib or brepocitinib. Guttman-Yassky et al. tested biomarkers of scalp alopecia areata in patients who participated in the clinical trial (31), presenting that although brepocitinib can directly inhibit IFN-γ signaling through JAK1/2 activation, ritlecitinib can indirectly affect IFN-γ production through inhibition of TEC family kinases. This is also consistent with our hypothesis. However, we did not analyse the difference in efficacy and safety between ritlecitinib and other JAK inhibitors in the treatment of AA this time because there were not enough high-quality literatures to include in the discussion.

Taken together, our study findings suggest that the addition of oral baricitinib or ruxolitinib to the drug regimen may be helpful for the treatment of AA. In terms of the therapeutic dose, an increasing trend with treatment duration was observed in the included studies. However, increasing the dose of JAK inhibitors may increase the risk of adverse effects (32). An increase in adverse reactions often causes patients to stop using medication. In addition, a previous meta-analysis (33, 34) showed that there is a close relationship between drug withdrawal and the recurrence of AA. Therefore, more dose-related studies are needed to clarify the maximum tolerated and therapeutic doses for treating patients with AA.

Safety outcomes demonstrated that the incidence of adverse effects was significantly lower with oral JAK inhibitors than with conventional steroid therapy. The reported adverse effects were mostly limited to mild symptoms and relatively manageable. The most common adverse events were upper respiratory tract infections, headache, acne, and biochemical abnormalities. Among them, the incidence of mild infection was the highest. Despite the selectivity of different JAK inhibitors in their pathways of action, there is considerable overlap in their safety profiles (35). Although the frequency of serious adverse events associated with oral JAK inhibitors was low in previous studies, we recommend that patients with AA treated with JAK inhibitors regularly check their liver function, complete blood count, and other laboratory indicators during treatment.

## 5 Limitations

This study compared the efficacy and safety of various JAK inhibitors in the treatment of AA through a network meta-analysis, providing a reference for clinical practice. However, it has the following limitations: (1) The sample size of most of the included studies (both prospective and retrospective studies) was small; therefore, the test power may have been insufficient. (2) There was heterogeneity in the included studies, including drug dosage, intervention course, and follow-up period, which may have led to bias in the results. (3) Most trials did not involve blinding of the subjects and interveners. In addition, there may have been selectivity bias during object selection. (4) Some of the included studies were still in the clinical trial stage at the time of compiling our findings, with further relevant clinical verification experiments needing completion. (5)

## 6 Conclusion

In conclusion, the clinical application of JAK inhibitors may be a new treatment strategy for AA. Oral JAK inhibitors are more effective than topical and sublingual JAK inhibitors in the treatment of AA. Oral baricitinib appears to be the best treatment option for patients with AA who desire a good response. In terms of side effects, oral JAK inhibitors are more tolerable in comparison to traditional steroid therapy. The adverse reactions to oral JAK inhibitors are mild and treatable; however, more high-quality, large-sample, multicenter, randomized controlled double-blind trials are needed to confirm these results.

## Data availability statement

The original contributions presented in the study are included in the article/**Supplementary Material.** Further inquiries can be directed to the corresponding author.

## Author contributions

DW and XZ conceived this meta-analysis. DW and YC selected the studies included in this meta-analysis. DW and YS performed statistical analyses. DW drafted this meta-analysis. BX edited language and grammar. All authors contributed to the article and approved the submitted version.
