# Supplementary material for: Efficacy and safety of different JAK inhibitors in the treatment of alopecia areata: a network meta-analysis
Source: Front Immunol. 2023 Apr 17;14:1152513. doi: 10.3389/fimmu.2023.1152513 (PMC10150113; doi:10.3389/fimmu.2023.1152513)
Supplement: Supplementary file 1 [file DataSheet_1.docx]

Supplementary Material

# Search formula for each database

# Search: ((((((((("Janus Kinase Inhibitors"[Mesh]) OR (Inhibitors, Janus Kinase)) OR (Kinase Inhibitors, Janus)) OR (JAK Inhibitors)) OR (Inhibitors, JAK)) OR (Janus Kinase Inhibitor)) OR (Inhibitor, Janus Kinase)) OR (Kinase Inhibitor, Janus)) OR (JAK Inhibitor)) OR (Inhibitor, JAK)

# Search: (((((((("tofacitinib" [Supplementary Concept]) OR (tasocitinib[Title/Abstract])) OR (tofacitinib citrate[Title/Abstract])) OR (Xeljanz[Title/Abstract])) OR (CP 690,550[Title/Abstract])) OR (CP690550[Title/Abstract])) OR (CP-690550[Title/Abstract])) OR (CP 690550[Title/Abstract])) OR (CP-690,550[Title/Abstract])

# Search: (((((((((((((((("ruxolitinib" [Supplementary Concept]) OR (3R)-3-cyclopentyl-3-(4-(7H-pyrrolo(2,3-d)pyrimidin-4-yl)pyrazol-1-yl)propanenitrile[Title/Abstract])) OR (ruxolitinib phosphate[Title/Abstract])) OR (ruxolitinib monophosphate[Title/Abstract])) OR (INCB-18424 phosphate[Title/Abstract])) OR (Jakavi[Title/Abstract])) OR (INCB018424 phosphate[Title/Abstract])) OR (INCB-018424 salt[Title/Abstract])) OR (Jakafi[Title/Abstract])) OR (ruxolitinib (as phosphate[Title/Abstract]))) OR (INCB-018424 phosphate[Title/Abstract])) OR (INCB-018424[Title/Abstract])) OR (INC-424[Title/Abstract])) OR (INCB-18424[Title/Abstract])) OR (INC424[Title/Abstract])) OR (INCB018424[Title/Abstract])) OR (INCA24[Title/Abstract])

# Search: (((((((((("baricitinib" [Supplementary Concept]) OR (3-azetidineacetonitrile, 1-(ethylsulfonyl)-3-(4-(7H-pyrrolo(2,3-d)pyrimidin-4-yl)-1H-pyrazol-1-yl)-[Title/Abstract])) OR (INCB-28050[Title/Abstract])) OR (Olumiant[Title/Abstract])) OR (baricitinib phosphate[Title/Abstract])) OR (baricitinib phosphate salt[Title/Abstract])) OR (3-azetidineacetonitrile, 1-(ethylsulfonyl[Title/Abstract])-3-(4-(7H-pyrrolo(2,3-d)pyrimidin-4-yl)-1H-pyrazol-1-yl)-, phosphate (1:1))) OR (INCB028050[Title/Abstract])) OR (INCB-028050[Title/Abstract])) OR (LY3009104[Title/Abstract])) OR (LY-3009104[Title/Abstract])

# Search: (("PF-06651600" [Supplementary Concept]) OR (1-(5-((7H-pyrrolo(2,3-d)pyrimidin-4-yl)amino)-2-methylpiperidin-1-yl)prop-2-en-1-one[Title/Abstract])) OR (Ritlecitinib[Title/Abstract])

# PUBMED

# (((((((((((((("Janus Kinase Inhibitors"[Mesh]) OR (Inhibitors, Janus Kinase)) OR (Kinase Inhibitors, Janus)) OR (JAK Inhibitors)) OR (Inhibitors, JAK)) OR (Janus Kinase Inhibitor)) OR (Inhibitor, Janus Kinase)) OR (Kinase Inhibitor, Janus)) OR (JAK Inhibitor)) OR (Inhibitor, JAK)) OR ((("PF-06651600" [Supplementary Concept]) OR (1-(5-((7H-pyrrolo(2,3-d)pyrimidin-4-yl)amino)-2-methylpiperidin-1-yl)prop-2-en-1-one[Title/Abstract])) OR (Ritlecitinib[Title/Abstract]))) OR ((((((((((("baricitinib" [Supplementary Concept]) OR (3-azetidineacetonitrile, 1-(ethylsulfonyl)-3-(4-(7H-pyrrolo(2,3-d)pyrimidin-4-yl)-1H-pyrazol-1-yl)-[Title/Abstract])) OR (INCB-28050[Title/Abstract])) OR (Olumiant[Title/Abstract])) OR (baricitinib phosphate[Title/Abstract])) OR (baricitinib phosphate salt[Title/Abstract])) OR (3-azetidineacetonitrile, 1-(ethylsulfonyl[Title/Abstract])-3-(4-(7H-pyrrolo(2,3-d)pyrimidin-4-yl)-1H-pyrazol-1-yl)-, phosphate (1:1))) OR (INCB028050[Title/Abstract])) OR (INCB-028050[Title/Abstract])) OR (LY3009104[Title/Abstract])) OR (LY-3009104[Title/Abstract]))) OR ((((((((((((((((("ruxolitinib" [Supplementary Concept]) OR (3R)-3-cyclopentyl-3-(4-(7H-pyrrolo(2,3-d)pyrimidin-4-yl)pyrazol-1-yl)propanenitrile[Title/Abstract])) OR (ruxolitinib phosphate[Title/Abstract])) OR (ruxolitinib monophosphate[Title/Abstract])) OR (INCB-18424 phosphate[Title/Abstract])) OR (Jakavi[Title/Abstract])) OR (INCB018424 phosphate[Title/Abstract])) OR (INCB-018424 salt[Title/Abstract])) OR (Jakafi[Title/Abstract])) OR (ruxolitinib (as phosphate[Title/Abstract]))) OR (INCB-018424 phosphate[Title/Abstract])) OR (INCB-018424[Title/Abstract])) OR (INC-424[Title/Abstract])) OR (INCB-18424[Title/Abstract])) OR (INC424[Title/Abstract])) OR (INCB018424[Title/Abstract])) OR (INCA24[Title/Abstract]))) OR ((((((((("tofacitinib" [Supplementary Concept]) OR (tasocitinib[Title/Abstract])) OR (tofacitinib citrate[Title/Abstract])) OR (Xeljanz[Title/Abstract])) OR (CP 690,550[Title/Abstract])) OR (CP690550[Title/Abstract])) OR (CP-690550[Title/Abstract])) OR (CP 690550[Title/Abstract])) OR (CP-690,550[Title/Abstract]))) AND (("Alopecia Areata"[Mesh]) OR (Alopecia Circumscripta[Title/Abstract]))

# 182 lectures

# WOS

# (TS=(Janus Kinase Inhibitors) OR AB=(Inhibitors, Janus Kinase OR Kinase Inhibitors, Janus OR JAK Inhibitors OR Inhibitors, JAK OR Janus Kinase Inhibitor OR Inhibitor, Janus Kinase OR Kinase Inhibitor, Janus OR JAK Inhibitor OR Inhibitor, JAK OR "PF-06651600" OR 1-5-7H-pyrrolo2,3-dpyrimidin-4-ylamino-2-methylpiperidin-1-ylprop-2-en-1-one OR Ritlecitinib OR "baricitinib" OR 3-azetidineacetonitrile, 1-ethylsulfonyl-3-4-7H-pyrrolo2,3-dpyrimidin-4-yl-1H-pyrazol-1-yl- OR INCB-28050 OR Olumiant OR baricitinib phosphate OR baricitinib phosphate salt OR 3-azetidineacetonitrile, 1-ethylsulfonyl-3-4-7H-pyrrolo2,3-dpyrimidin-4-yl-1H-pyrazol-1-yl-, phosphate 1:1 OR INCB028050 OR INCB-028050 OR LY3009104 OR LY-3009104 OR "ruxolitinib" OR 3R-3-cyclopentyl-3-4-7H-pyrrolo2,3-dpyrimidin-4-ylpyrazol-1-ylpropanenitrile OR ruxolitinib phosphate OR ruxolitinib monophosphate OR INCB-18424 phosphate OR Jakavi OR INCB018424 phosphate OR INCB-018424 salt OR Jakafi OR ruxolitinib as phosphate OR INCB-018424 phosphate OR INCB-018424 OR INC-424 OR INCB-18424 OR INC424 OR INCB018424 OR INCA24 OR "tofacitinib" OR tasocitinib OR tofacitinib citrate OR Xeljanz OR CP 690,550 OR CP690550 OR CP-690550 OR CP 690550 OR CP-690,550)) AND (TS=(Alopecia Areata) OR AB=(Alopecia Circumscripta))

# 249lectures

# Cochrane

# Search Name:

# Date Run: 25/01/2023 18:26:13

# Comment:

# Warning: Problems were found with one or more of your search lines (specific lines are identified below). For best results, you should review and edit the search lines indicated.

# ID Search Hits

# #1 MeSH descriptor: [Alopecia Areata] explode all trees 355

# #2 (Alopecia Areata or Alopecia Circumscripta):ti,ab,kw (Word variations have been searched) 682

# #3 #1 or #2 682

# #4 MeSH descriptor: [Janus Kinase Inhibitors] explode all trees 105

# #5 (Janus Kinase Inhibitors or Inhibitors, Janus Kinase or Kinase Inhibitors, Janus or JAK Inhibitors or Inhibitors, JAK or Janus Kinase Inhibitor or Inhibitor, Janus Kinase or Kinase Inhibitor, Janus or JAK Inhibitor or Inhibitor, JAK):ti,ab,kw (Word variations have been searched) 2136

# #6 #4 or #5 2136

# #7 #3 and #6 34

# 34 lectures

# EMBASE

# Embase Session Results

# No.QueryResults

# 394

# #5

# #3 AND #4

# 14,427

# #4

# 'janus kinase inhibitors':ab,ti OR 'inhibitors, janus kinase':ab,ti OR 'kinase inhibitors, janus':ab,ti OR 'jak inhibitors':ab,ti OR 'inhibitors, jak':ab,ti OR 'janus kinase inhibitor':ab,ti OR 'inhibitor, janus kinase':ab,ti OR 'kinase inhibitor, janus':ab,ti OR 'jak inhibitor':ab,ti OR 'inhibitor, jak':ab,ti OR 'pf-06651600':ab,ti OR '1 5 7h pyrrolo2,3 dpyrimidin 4 ylamino 2 methylpiperidin 1 ylprop 2 en 1 one':ab,ti OR ritlecitinib:ab,ti OR 'baricitinib':ab,ti OR '3-azetidineacetonitrile, 1-ethylsulfonyl-3-4-7h-pyrrolo2,3-dpyrimidin-4-yl-1h-pyrazol-1-yl-':ab,ti OR 'incb 28050':ab,ti OR olumiant:ab,ti OR 'baricitinib phosphate':ab,ti OR 'baricitinib phosphate salt':ab,ti OR '3-azetidineacetonitrile, 1-ethylsulfonyl-3-4-7h-pyrrolo2,3-dpyrimidin-4-yl-1h-pyrazol-1-yl-, phosphate 1:1':ab,ti OR incb028050:ab,ti OR 'incb 028050':ab,ti OR ly3009104:ab,ti OR 'ly 3009104':ab,ti OR 'ruxolitinib':ab,ti OR '3r 3 cyclopentyl 3 4 7h pyrrolo2,3 dpyrimidin 4 ylpyrazol 1 ylpropanenitrile':ab,ti OR 'ruxolitinib phosphate':ab,ti OR 'ruxolitinib monophosphate':ab,ti OR 'incb-18424 phosphate':ab,ti OR jakavi:ab,ti OR 'incb018424 phosphate':ab,ti OR 'incb-018424 salt':ab,ti OR jakafi:ab,ti OR 'ruxolitinib as phosphate':ab,ti OR 'incb-018424 phosphate':ab,ti OR 'incb 018424':ab,ti OR 'inc 424':ab,ti OR 'incb 18424':ab,ti OR inc424:ab,ti OR incb018424:ab,ti OR inca24:ab,ti OR 'tofacitinib':ab,ti OR tasocitinib:ab,ti OR 'tofacitinib citrate':ab,ti OR xeljanz:ab,ti OR cp690550:ab,ti OR 'cp 690550':ab,ti OR 'cp 690,550':ab,ti

# 6,737

# #3

# 'alopecia areata':ab,ti OR 'alopecia circumscripta':ab,ti

# 394 lectures
